# Presence of chemotherapy-induced toxicity predicts improved survival in patients with localised extremity osteosarcoma treated with doxorubicin and cisplatin: A report from the European Osteosarcoma Intergroup

**DOI:** 10.1016/j.ejca.2011.09.012

**Published:** 2012-03

**Authors:** Anne McTiernan, Rachel C. Jinks, Matthew R. Sydes, Barbara Uscinska, Jane M. Hook, Martine van Glabbeke, Vivien Bramwell, Ian J. Lewis, Antonie H.M. Taminiau, Marianne A. Nooij, Pancras C.W. Hogendoorn, Hans Gelderblom, Jeremy S. Whelan

**Affiliations:** a Department of Oncology, University College Hospital, London, UK; b Cancer Group, Medical Research Council Clinical Trials Unit, London, UK; c Soft Tissue and Bone Sarcoma Group, European Organization for Research and Treatment of Cancer Data Center, Brussels, Belgium; d Department of Medical Oncology, Tom Baker Cancer Centre, Calgary, Alberta, Canada; e Medical Director, Alder Hey Children’s NHS Foundation Trust, Liverpool, UK; f Department of Orthopaedic Surgery, Leiden University Medical Center, Leiden, The Netherlands; g Department of Clinical Oncology, Leiden University Medical Center, Leiden, The Netherlands; h Department of Pathology, Leiden University Medical Center, Leiden, The Netherlands

**Keywords:** Osteosarcoma, Toxicity, Survival, Chemotherapy

## Abstract

**Aim:**

Chemotherapy-induced toxicity is an independent prognostic indicator in several cancers. We aimed to determine whether toxicity was related to survival and histological response in high-grade localised extremity osteosarcoma. We undertook a retrospective analysis of patients treated within three consecutive randomised controlled trials (RCTs) of the European Osteosarcoma Intergroup.

**Methods:**

Between 1982 and 2002, 533 patients were randomised to six cycles of doxorubicin 75 mg/m^2^ and cisplatin 100 mg/m^2^. Toxicity data were collected prospectively and graded according to the World Health Organisation (WHO) criteria. Standard univariate and multivariate models were constructed to examine the relationship between reported toxicity, survival, and histological response.

**Results:**

Five- and 10-year overall survival was 57% (95% confidence interval (CI) 52–61%) and 53% (49–58%), respectively. Grades 3–4 oral mucositis (hazard ratio (HR) 0.51, 95% CI 0.29–0.91), grades 1–2 nausea/vomiting (HR 0.37, 95% CI 0.16–0.85), grades 1–2 thrombocytopenia (HR 0.49, 95% CI 0.27–0.87), good histological response (HR 0.42, 95% CI 0.27–0.65), and distal tumour site (HR 0.45, 95% CI 0.28–0.71) were associated with improved survival in multivariate analysis. The only factors that were independently associated with histological response were older age (odds ratio (OR) 0.18, 95% CI 0.04–0.72) and chondroblastic tumour (OR 0.28, 95% CI 0.10–0.77), both being associated with a significantly lower chance of achieving a good response.

**Conclusion:**

Chemotherapy-induced toxicity predicts survival in patients with localised extremity osteosarcoma. Investigation of the pharmacogenomic mechanisms of constitutional chemosensitivity underlying these observations will present opportunities for personalising treatment and could lead to improved outcomes.

## 1 Introduction

Osteosarcoma is the commonest primary bone sarcoma affecting young people. The prognosis of patients with high-grade localised extremity osteosarcoma improved dramatically with the introduction of multi-disciplinary treatment (surgical resection in conjunction with perioperative multi-agent chemotherapy) but over the past two decades there have been no further improvements in survival.

Histological response to pre-operative chemotherapy is strongly related to the outcome, with patients who achieve a good histological response having a better prognosis than those who do not.^1–4^ However, identifying other factors that are reliably prognostic for survival or predictive of response to treatment has been problematic and, although evidence for the influence of several other factors, including histological subtype,^5^ has been reported, none routinely influence practice.

In other cancers, chemotherapy-induced toxicity has been shown to be an independent prognostic indicator, with those patients who report greater toxicity also having improved survival. The strongest association has been with myelosuppression. Prognostic effects of chemotherapy-induced neutropenia have been demonstrated in breast,^7^ gastric,^6^ lung^7,8^ and ovarian cancer^9^ in adults; and in children and adolescents receiving maintenance treatment for acute lymphoblastic leukaemia.^10,11^ Chemotherapy-induced lymphopenia has been shown to be prognostic in advanced breast cancer, soft-tissue sarcoma, and diffuse large B-cell lymphoma.^12^ If a similar association between toxicity and either histological response or survival was found in patients with osteosarcoma, an understanding of the underlying genetic and other mechanisms which may explain this constitutional chemosensitivity could lead to the testing and development of therapeutic strategies to exploit or circumvent these phenomena, with the prospect of greater individualisation of treatment and improved outcomes.

The European Osteosarcoma Intergroup (EOI) has completed three randomised controlled trials (RCTs), involving over 1000 patients with localised extremity osteosarcoma. The same ‘standard’ treatment arm was used in all three, creating a uniquely large cohort treated in a standard manner and followed prospectively.

To explore whether chemotherapy-induced toxicity was associated with outcome in patients with high-grade osteosarcoma, we undertook a retrospective analysis exploring factors relating to survival and histological response in this cohort.

## 2 Patients and methods

### 2.1 Patients

**Fig. 1:**
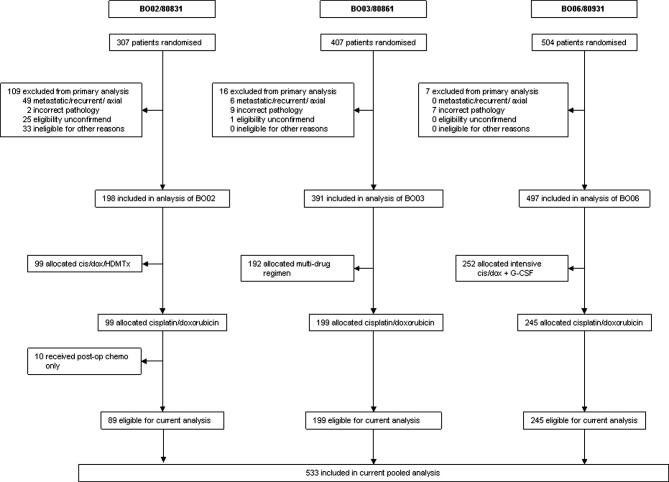
CONSORT diagram.

Between 1982 and 2002, three consecutive EOI chemotherapy RCTs (MRC BO02/EORTC80831, BO03/80861, BO06/80931) randomised 1067 patients. In each, one arm of the randomisation was a ‘standard’ treatment: six 3-weekly cycles of doxorubicin 25 mg/m^2^/d for 3 d, plus cisplatin 100 mg/m^2^ as a continuous 24-h infusion on day 1. Hydration schedules were protocol-specified but other supportive care, including antiemetic regimens, was in accordance with the local practice at trial sites. Surgery was scheduled after either three (BO02 and BO03) or two (BO06) cycles. Full details of each trial are reported elsewhere.^4,13,14^ Ninety-nine patients were randomised to standard treatment in BO02 (1983–1986),^13^ 199 in BO03 (1986–1991),^4^ and 245 in BO06 (1993–2002).^14^ Ten patients electively treated with post-operative chemotherapy alone in BO02 were excluded from this study, in line with previous reports from this series.^5,13,15^ In total, 533 patients were included in the current combined analysis (Fig. 1).

Patients aged ⩽40 years with histologically proven, high-grade, localised extremity osteosarcoma, and adequate renal and cardiac function were eligible. Patients who had received prior chemotherapy or had a previous malignancy were ineligible. Ethics approval was granted at all institutions, and written informed consent obtained from the patient or parent, in accordance with the local regulatory guidelines. Patients were randomised within 35 d after diagnostic biopsy. The resected specimen was examined histologically to assess response to pre-operative chemotherapy. Good histological response was defined as ⩾90% necrosis in the tumour resected. Both the diagnostic pathology and response assessment were reviewed by the EOI pathology sub-committee.

### 2.2 Data

In each RCT, toxicity data were collected prospectively at each cycle of chemotherapy using standardised case-report forms and graded at site according to the World Health Organisation (WHO) criteria^16^ for haematological toxicity, infection, mucositis, nausea/vomiting, neurological toxicity and cardiac toxicity. Renal toxicity was not specifically recorded in BO06 so this factor was not included in this combined analysis. The worst grade of toxicity for each patient in any pre-operative cycle was used in analyses of histological response, and the worst grade of toxicity per-patient for all cycles was used in the analyses of overall and progression-free survival. Data for leucopenia and thrombocytopenia were not available for the first trial (BO02). Data on non-haematological toxicity were available for 517/533 (97%) patients. Data on histological response were available for 351/533 (66%) patients.

### 2.3 Statistical analysis

This was a retrospective analysis, carried out on an intention-to-treat basis, in which data from the original three trials were combined and analysed as one data-set. Analyses were undertaken to examine prognostic factors for overall survival, progression-free survival (PFS) and histological response. Baseline characteristics examined included: collaborative group, geographical location, age, gender, primary site (bone affected), proximal versus distal tumour (proximal defined as a proximal tumour of the humerus or femur; distal as all other sites), and histological subtype. Treatment-related factors included type and timeliness of surgery, histological response to pre-operative chemotherapy and chemotherapy-induced toxicity. A two-sided significance level of 5% was adopted. Stata 9 (StataCorp, College Station, TX) was used for the analysis.

Analyses of factors influencing overall survival and PFS used standard time-to-event methodology (survival analysis).^17^ Median follow-up was calculated by reverse censoring on overall survival. Overall survival was timed until death (from any cause) or patients were censored at the date of last follow-up if death had not occurred. PFS was timed until date of first event (local or metastatic disease progression or death but excluding apparent progression of local disease before primary surgery), or censoring occurred at date of last follow-up. Fourteen patients had apparent progression of local disease before primary surgery but these events were not included in recognition that early clinical distinction between response and progression is unreliable in osteosarcoma; the patients were included in the survival analyses. The relative risks of each factor are summarised using hazard ratios (HR) from univariate and multivariate Cox regression models. HRs are expressed relative to patients in the baseline category of the factor of interest; so an HR <1.0 indicates a lower risk of the event compared to the baseline category. Variables were considered to be nominal. For the univariate models, survival was measured from date of randomisation, or from date of surgery for factors measured at surgery (histological response, type of surgery). In the multivariate analyses, survival was measured from the date of surgery.

The impact of factors on histological response was examined using univariate and multivariate logistic regression models, and expressed by odds ratios (OR). An OR >1.0 indicates a greater chance of achieving a good response, compared to the baseline category.

For both the Cox and logistic regression models, patients with missing data for the variable of interest were excluded from that particular univariate analysis. Only patients with data available for all factors were incorporated in the multivariate analyses. All models were stratified by trial.

## 3 Results

### 3.1 Patient characteristics

Patient characteristics are shown in Table 1. They were broadly similar across all three studies, although the proportion of males and chondroblastic osteosarcomas was lower in BO02 (Supplementary data). Median age was 15 years (Inter Quartile Range (IQR), 12–19). Median follow-up was 9.9 years (5.2–14.8); being 17.9 years (16.8–19.2) for BO02; 12.7 years (11.1–14.7) for BO03; and 5.0 years (3.0–7.0) for BO06.

### 3.2 Treatment

Treatment received is shown in Table 2. The proportion of patients completing six cycles of chemotherapy as scheduled was 80%. Six percent of patients stopped treatment due to progressive disease. Patients were slightly more likely to be reported as stopping early for excessive toxicity within BO02 (Supplementary Data). Most toxicities were common with >90% of patients reporting nausea/vomiting (504/519) and leucopenia (409/437); and >60% reporting mucositis (338/518), infection (321/517) and thrombocytopenia (342/427).

Patients were more likely to undergo an amputation in BO02 (44%) compared to the later studies (26% within each) (Supplementary data). The overall proportion of patients achieving a good histological response was 35%.

### 3.3 Overall survival

**Fig. 2:**
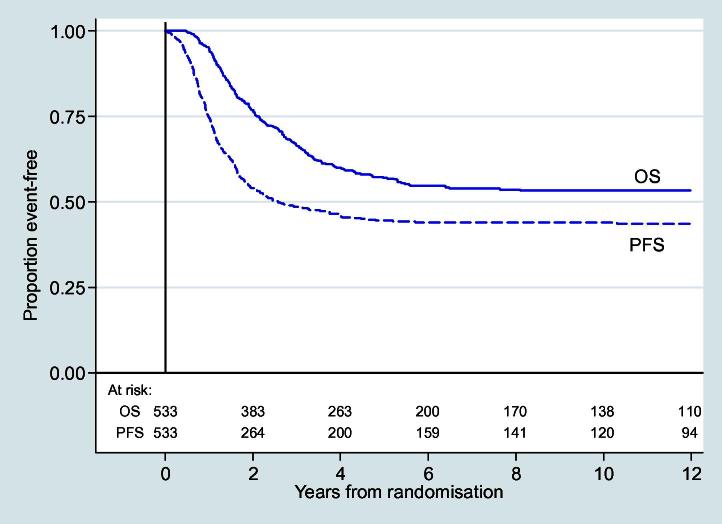
Overall and progression-free survival.

The 5- and 10-year overall survival for all patients was 57% (95% confidence interval (CI) 52–61%) and 53% (49–58%), respectively (Fig. 2). In total, 227 deaths were observed.

Univariate and multivariate analyses for overall survival are shown in Table 3.

#### 3.3.1 Factors at diagnosis

No differences in survival were observed according to collaborative group, geographical location, or age. Female gender was associated with improved survival in univariate analysis, but the effect size decreased in the multivariate model. Primary site was a prognostic indicator, with the humerus having the poorest survival (5-year overall survival 41%, 95% CI 27–55%), and the tibia the best (66%, 57–73%). Patients with proximal tumours had poorer survival compared to those with distal tumours (40%, 28–52% versus 60%, 55–64%). There was no statistically significant difference in survival between the main histological subtypes but the small (*n* = 22) heterogeneous group categorised as ‘other’ appeared to have the poorest prognosis.

#### 3.3.2 Treatment-related factors

Histological response was prognostic for survival, with patients who had a good histological response having a 5-year overall survival of 71% (95% CI 61–78%) versus 47% (38–51%) for those with a poor response. There was no evidence of a difference in survival according to the type of surgery or for patients whose surgery took place more than 10 d later than specified. In univariate analysis, early surgery was associated with worse 5-year survival, being 42% (24–59%), versus 55% (48–62%) for patients whose surgery was on-time (HR 1.67, 95% CI 0.98–2.87) but this effect disappeared in multivariate analysis (0.91, 0.28–3.01).

**Fig. 3:**
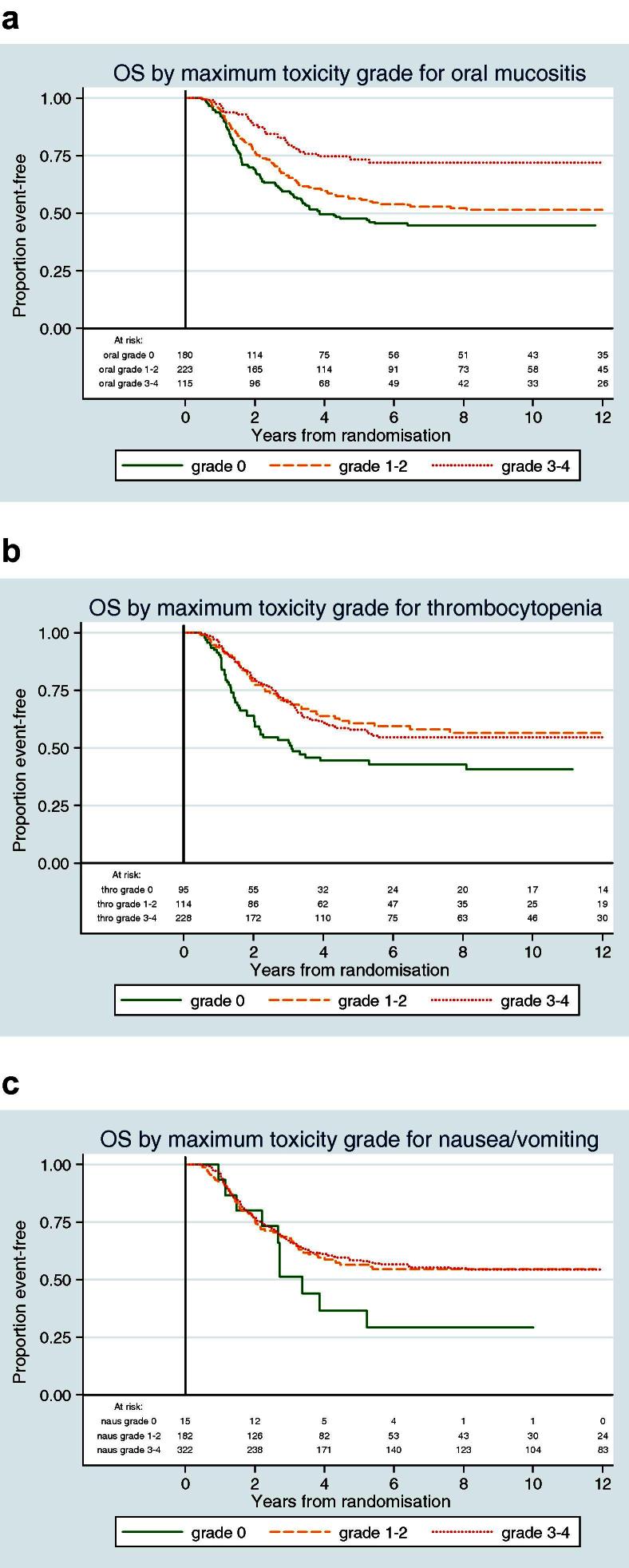
Overall survival, according to chemotherapy related toxicity.

Chemotherapy-induced toxicities were prognostic for overall survival, with the presence of greater toxicity generally being associated with better survival. In univariate analysis, toxicities with the greatest effect on survival were grades 3–4 oral mucositis (Fig. 3), grades 1–2 peripheral neuropathy and thrombocytopenia of any grade (Fig. 3). Grades 1–4 nausea/vomiting identified a group of patients with some evidence of improved survival (Fig. 3). Within multivariate analysis, grades 3–4 oral mucositis, grades 1–2 nausea/vomiting and grades 1–2 thrombocytopenia were associated with improved survival. No evidence of an association with survival was found with leucopenia, infection or cardiac toxicity (experienced during chemotherapy). Only one patient experienced grades 3–4 peripheral neuropathy so no results could be drawn for this category. Stratifying by whether or not patients had completed all six cycles of chemotherapy had no impact on the toxicity-related factors that were statistically significant for an impact on survival (data not shown).

### 3.4 Progression-free survival

Five- and 10-year PFS was 44% (95% CI 40–49%) and 44% (39–48%), respectively (Fig. 2), with 284 events observed. The univariate and multivariate analyses for PFS are shown in Table 3. Primary site and histological response to pre-operative chemotherapy were also strongly associated with PFS but there was no evidence of an association with gender or timeliness of surgery. Within multivariate analysis, patients with anaplastic tumours (16/328) appeared to have a better PFS compared to other histological subtypes. Chemotherapy-induced toxicity was also a prognostic factor for PFS. Grades 1–4 oral mucositis, grades 1–2 nausea/vomiting, grades 1–2 peripheral neuropathy, thrombocytopenia of any grade and grades 3–4 leucopenia were associated with improved PFS in univariate analysis; on multivariate analysis, the effect of leucopenia and grades 1–2 oral mucositis lessened.

### 3.5 Histological response

Older age and chondroblastic tumours were associated with a significantly lower chance of reporting a good histological response (age ⩾26 years, OR 0.18, 95% CI 0.04–0.72; chondroblastic tumours, 0.28, 0.10–0.77) (Supplementary data). No association was found with chemotherapy-induced toxicity, or any other factor.

## 4 Discussion

This paper uniquely explores the association between chemotherapy-induced toxicity and outcome in patients with osteosarcoma. Chemotherapy-induced toxicity was associated with improved overall and progression-free survival. It did not predict an increased likelihood of having a good histological response to pre-operative chemotherapy, however, although this was itself prognostic for survival. Although the results are derived from a cohort of patients treated with doxorubicin and cisplatin alone, these agents remain central to all widely used regimens for osteosarcoma. The incorporation of other agents such as methotrexate into current regimens may alter the spectrum of toxicities experienced by patients and, while there is no empirical reason to conclude that the addition of methotrexate would erase the predictive value of chemotherapy-induced toxicity, further study is warranted.

In studies exploring chemotherapy-induced toxicity in other cancers, myelosuppression has been associated with improved outcome raising the possibility of using this as an indicative marker for chemotherapy efficacy.^6,12,18^ In contrast, we found that non-haematological toxicities appear to be a better indicator of survival in osteosarcoma. Although there was a trend for improved survival in patients experiencing myelosuppression, this only remained statistically significant for thrombocytopenia. It is likely that patients with osteosarcoma experience a higher incidence of grades 3–4 myelosuppression than patients with other tumours, due to more aggressive chemotherapy, thus diminishing its relative association with survival. In addition, although not routinely recorded in these trials, G-CSF was permitted on a per-patient basis, which may have reduced the overall incidence of neutropenia. Increased nausea and vomiting was associated with a trend for increased survival. The reported incidence of this toxicity decreased in later studies, probably due to better antiemetic prophylaxis; hence it is likely that its prognostic sensitivity has now decreased.

In osteosarcoma, little is known about the influence of inter-patient variations in chemotherapy metabolism on its tolerability or effectiveness. Wide inter- and intra-variability in serum methotrexate levels has been observed following high-dose methotrexate (8–12 g/m^2^).^19^ Higher serum methotrexate levels have been linked with better survival^20,21^ but results have been inconsistent, with others finding no difference or even poorer survival, with peak concentrations >1500 μM.^22^ There are no systematic studies on pharmacogenetic markers linked to cisplatin or doxorubicin metabolism. However, studies to identify relevant polymorphisms in patients with osteosarcoma are now underway.^23^

This analysis has some limitations. Haematological toxicity data were not available for the earliest trial and histological response to chemotherapy was missing for 182 patients (34%). Despite this being, for osteosarcoma, a large cohort, some of the categories in the analyses involved small numbers, in particular: histological subtypes other than common-type or chondroblastic, and grades 3–4 neurological and cardiac toxicity. These were retrospective exploratory analyses which are at risk of statistical anomalies. Additionally, other factors of potential interest were not routinely recorded, including lymphocyte count, alkaline phosphatase and lactate dehydrogenase levels, nephrotoxicity and tumour size. Only acute cardiac toxicity was recorded and it remains possible that sub-acute or late cardiac toxicity may be associated with survival. Although the toxicity data was recorded using a standardised grading system, there is a subjective element to assessing non-haematological toxicity and we could not control for variation between individual investigators. Early lymphocyte recovery following chemotherapy has been suggested to be prognostic in a small study of children with osteosarcoma,^24^ with similar findings reported in Ewing’s sarcoma,^25^ and this observation warrants investigation in future prospective studies.

Although the main chemotherapy protocol remained constant, other aspects of osteosarcoma treatment and supportive care changed over the 20 years that these studies cover. In the later RCTs, limb-sparing surgery was performed more often and the use of more effective antiemetics has already been cited as a probable reason for a decrease in the reported incidence of chemotherapy-induced nausea and vomiting. Changes in practice are accounted for by the modelling techniques used in this analysis; however, other changes are more problematic. For example, the decreased prevalence of common-type osteosarcoma and increased proportion of chondroblastic tumours over time may be due to improvements in pathology. Such changes in reporting practices are not always easily identifiable, and are difficult to account for in retrospective analyses.

These data for osteosarcoma are consistent with those from other cancers in showing that the presence of some chemotherapy-induced toxicities may predict improved survival. Doxorubicin and cisplatin remain an integral part of treatment together with high-dose methotrexate in the current EURAMOS-1 trial (NCT00134030).^26^ A similar association between toxicity and survival may exist with this regimen and can be explored when the trial matures. In the absence of new chemotherapy agents, novel approaches to optimising treatment are still needed and the prospect of individualisation of treatment through isotoxic regimens is an intriguing prospect that could be explored in future clinical trials. However, this is complicated by our finding that non-haematological, rather than haematological, toxicity was the strongest predictor of survival. Due to the subjective elements of evaluating non-haematological toxicity, and the effects of improvements in supportive care, further elucidation of the underlying mechanisms which account for individual variation in toxicity and how these might link to mechanisms of tumour sensitivity and resistance are necessary before reliable recommendations can be made. For now, lack of toxicity may provide early evidence of reduced constitutional chemosensitivity, and predict poorer outcome in patients with osteosarcoma.

Osteosarcoma predominantly affects adolescents and young adults (AYAs) and age-group specific differences in pharmacodynamics have been postulated to be one of the reasons that survival in AYAs with cancer has failed to improve at the same rate as that of children and older adults. There are few data directly addressing chemotherapy dosing in this age-group. The next RCT from the EURAMOS group intends to address these issues and to capture individual’s genetic information. Our data encourage the search for genetic predictors of individual toxicity and sensitivity to chemotherapy in osteosarcoma.

## Funding

This work was supported by the Medical Research Council and was in part carried out at the University College Hospital NHS Foundation Trust, which receives funding through the National Institute of Health Research Comprehensive Biomedical Research Centre programme.

## Contributors

BU, MvG, VB, IJL, AHMT, MAN, PCWH and HG were involved in the design, conduct, analysis and interpretation of the source trials and the conception of this analysis. JSW and MRS were also responsible for the study concept. This study was designed by AMcT, JSW, MRS and RCJ. Data analysis was done by RCJ and MRS. Data interpretation was done by AMcT, JW, MRS, and RCJ. The report was written by AMcT, JMH, MRS, RCJ, and JSW. All authors (except for MvG) commented on and approved the report.

## Conflict of interest statement

None declared.

## Figures and Tables

**Table 1:** Patient demographics and clinical characteristics.

	Total (*n *= 533)
*Collaborative group*	
MRC	334 (63%)
EORTC	199 (37%)

*Geographical location*	
UK/Ireland	303 (57%)
Mainland Europe	136 (26%)
Others^a^	94(18%)

*Age at randomisation*	
Median (years)	15 (12–19)
Min–Max	3–40
0–10 years	93 (17%)
11–15 years	181 (34%)
16–20 years	172 (32%)
21–25 years	52 (10%)
⩾26 years	35 (7%)
Missing	0 (n/a)

*Sex*	
Male	323 (61%)
Female	207 (39%)
Missing	3 (n/a)

*Site of tumour*	
Femur	307 (58%)
Tibia	136 (26%)
Fibula	28 (5%)
Humerus	52 (10%)
Radius	6 (1%)
Missing	4 (n/a)

*Location of tumour*	
Proximal^b^	69 (13%)
Distal^c^	458 (87%)
Missing	6 (n/a)

*Classification of sarcoma*	
Common-type	331 (66%)
Chondroblastic	55 (11%)
Fibroblastic	38 (8%)
Osteoclast rich	6 (1%)
Anaplastic	16 (3%)
Small cell	6 (1%)
Telangiectatic	26 (5%)
Other	22 (4%)
Missing	33 (n/a)

*Trial*	
BO02/80831	89 (17%)
BO03/80931	199 (37%)
BO06/80861	245 (46%)

Data are number (%) or median (IQR).

a Other geographical location = South America, South Africa, Saudi Arabia, Canada, New Zealand.

b Proximal = proximal humerus/femur.

c Distal = all other sites.

**Table 2:** Treatment details, response and toxicity.

	Total (*n *= 533)
*Reason off-study*	
Treatment completed	423 (80%)
Disease progression	29 (6%)
Excessive toxicity	35 (7%)
Treatment refusal	16 (3%)
Other	25 (5%)
Missing	5 (n/a)

*Type of surgery*	
Amputation	147 (29%)
Limb salvage	359 (71%)
Missing	25 (n/a)

*Histological response*	
Poor	228 (65%)
Good	123 (35%)
Missing	182 (n/a)

*Max oral mucositis grade*	
Grade 0	180 (35%)
Grades 1–2	223 (43%)
Grades 3–4	115 (22%)
Missing	15 (n/a)

*Max nausea/vomiting grade*	
Grade 0	15 (3%)
Grades 1–2	182 (35%)
Grades 3–4	322 (62%)
Missing	14 (n/a)

*Max cardiac grade*^a^	
Grade 0	464 (90%)
Grades 1–2	48 (9%)
Grades 3–4	6 (1%)
Missing	15 (n/a)

*Max infection grade*	
Grade 0	196 (38%)
Grades 1–2	201 (39%)
Grades 3–4	120 (23%)
Missing	16 (n/a)

*Max neurological grade*	
Grade 0	458 (89%)
Grades 1–2	58 (11%)
Grades 3–4	1 (0%)
Missing	16 (n/a)

*Max leucopenia grade*	
Grade 0	28 (6%)
Grades 1–2	66 (15%)
Grades 3–4	343 (79%)
Missing	96 (n/a)

*Max thrombocytopenia grade*	
Grade 0	95 (22%)
Grades 1–2	114 (26%)
Grades 3–4	228 (52%)
Missing	96 (n/a)

Data are number (%).

a As recorded during chemotherapy only.

**Table 3:** Univariate and multivariate Cox models for overall and progression-free survival.

	*n*	Overall survival				Progression-free survival			
		Univariate models		Multivariate model (*n *= 328)		Univariate models		Multivariate model (*n *= 328)	
		Hazard ratio (95% CI)	*p*-Value	Hazard ratio (95% CI)	*p*-Value	Hazard ratio (95% CI)	*p*-Value	Hazard ratio (95% CI)	*p*-Value
*Year of randomisation*^a^									
Each additional year from 1983	533	1.01 (0.98–1.03)	0.599			1.02 (1.00–1.04)	0.052		
*Collaborative group*	533								
MRC		1.00		1.00		1.00		1.00	
EORTC		1.03 (0.78–1.35)	0.856	0.53 (0.19–1.45)	0.217	0.92 (0.72–1.18)	0.529	0.50 (0.21–1.18)	0.112
*Geographical location*	533								
UK/Ireland		1.00		1.00		1.00		1.00	
Mainland Europe		1.09 (0.79–1.49)	0.599	1.44 (0.49–4.24)	0.510	0.91 (0.68–1.22)	0.536	1.48 (0.57–3.89)	0.422
Other		1.02 (0.70–1.49)	0.905	1.23 (0.52–2.94)	0.638	0.93 (0.67–1.29)	0.673	1.16 (0.55–2.41)	0.697
*Age group*	533								
⩽10 years		1.00		1.00		1.00			
11–15 years		1.07 (0.72–1.61)	0.730	1.08 (0.64–1.83)	0.777	1.20 (0.85–1.71)	0.305	1.30 (0.83–2.05)	0.254
16–20 years		1.17 (0.78–1.75)	0.448	1.27 (0.74–2.18)	0.382	1.14 (0.80–1.64)	0.471	1.24 (0.76–2.02)	0.381
21–25 years		0.92 (0.53–1.61)	0.772	0.75 (0.34–1.64)	0.467	0.92 (0.56–1.51)	0.748	0.87 (0.45–1.69)	0.676
⩾26 years		1.53 (0.87–2.67)	0.136	2.03 (0.97–4.24)	0.061	1.22 (0.72–2.06)	0.468	1.29 (0.61–2.69)	0.505
*Gender*	530								
Male		1.00		1.00		1.00		1.00	
Female		0.73 (0.55–0.97)	0.028	0.68 (0.45–1.01)	0.054	0.82 (0.64–1.04)	0.101	0.80 (0.57–1.12)	0.189
*Site of tumour*^a^	529								
Femur		1.00				1.00			
Tibia		0.68 (0.49–0.96)	0.026			0.69 (0.51–0.93)	0.014		
Fibula		0.94 (0.52–1.70)	0.842			0.92 (0.54–1.56)	0.749		
Humerus		1.57 (1.07–2.32)	0.023			1.81 (1.27–2.57)	0.001		
Other		0.53 (0.13–2.14)	0.371			0.66 (0.21–2.06)	0.471		
*Location of tumour*	527								
Proximal		1.00		1.00		1.00		1.00	
Distal		0.54 (0.39–0.75)	<0.001	0.45 (0.28–0.71)	0.001	0.49 (0.36–0.66)	<0.001	0.34 (0.23–0.52)	<0.001
*Type of osteosarcoma*	500								
Common-type		1.00		1.00		1.00		1.00	
Chondroblastic		1.13 (0.73–1.74)	0.575	0.75 (0.42–1.36)	0.343	1.38 (0.96–1.99)	0.081	0.87 (0.54–1.40)	0.575
Fibroblastic		1.16 (0.72–1.89)	0.545	1.49 (0.78–2.86)	0.226	1.11 (0.71–1.76)	0.643	1.61 (0.88–2.95)	0.119
Anaplastic		0.84 (0.37–1.91)	0.682	0.39 (0.12–1.34)	0.137	0.74 (0.35–1.58)	0.435	0.32 (0.11–0.95)	0.040
Telangiectatic		1.23 (0.64–2.38)	0.530	0.98 (0.45–2.17)	0.967	0.84 (0.45–1.57)	0.590	0.77 (0.37–1.63)	0.500
Other		1.77 (1.08–2.90)	0.023	1.88 (1.01–3.50)	0.046	1.64 (1.05–2.57)	0.029	1.38 (0.78–2.42)	0.265
*Oral Mucositis*	518								
Grade 0		1.00		1.00		1.00		1.00	
Grades 1–2		0.75 (0.57–1.00)	0.053	1.01 (0.67–1.53)	0.946	0.70 (0.53–0.90)	0.007	0.96 (0.66–1.38)	0.809
Grades 3–4		0.38 (0.25–0.57)	<0.001	0.51 (0.29–0.91)	0.023	0.48 (0.34–0.68)	<0.001	0.61 (0.37–0.99)	0.046
*Nausea and vomiting*	519								
Grade 0		1.00		1.00					
Grades 1–2		0.60 (0.31–1.16)	0.129	0.37 (0.16–0.85)	0.020	0.55 (0.30–1.00)	0.049	0.35 (0.16–0.76)	0.008
Grades 3–4		0.60 (0.31–1.14)	0.117	0.53 (0.23–1.20)	0.127	0.61 (0.34–1.10)	0.103	0.49 (0.22–1.05)	0.068
*Infection*	517								
Grade 0		1.00		1.00		1.00		1.00	
Grades 1–2		0.84 (0.62–1.14)	0.269	0.91 (0.58–1.42)	0.666	0.90 (0.68–1.19)	0.459	1.07 (0.72–1.60)	0.733
Grades 3–4		0.71 (0.49–1.03)	0.072	0.90 (0.52–1.56)	0.715	0.85 (0.62 –1.17)	0.313	1.44 (0.89–2.32)	0.135
*Cardiac*	518								
Grade 0		1.00		1.00		1.00		1.00	
Grades 1–2		0.84 (0.52–1.36)	0.481	0.85 (0.44–1.68)	0.648	0.99 (0.66–1.49)	0.959	1.44 (0.83–2.48)	0.193
Grades 3–4		0.71 (0.18–2.90)	0.636	1.02 (0.13–8.07)	0.988	0.67 (0.21–2.12)	0.501	1.04 (0.23–4.65)	0.958
*Neurological*	517								
Grade 0		1.00		1.00		1.00		1.00	
Grades 1–2		0.51 (0.31–0.85)	0.010	0.81 (0.43–1.52)	0.513	0.45 (0.28–0.72)	0.001	0.56 (0.32–0.99)	0.044
Grades 3–4		1.73 (0.24–12.42)	0.587	0.71 (0.07–7.63)	0.780	3.75 (0.52–27.22)	0.192	2.16 (0.21–22.72)	0.520
*Leucopenia*	437								
Grade 0		1.00		1.00		1.00		1.00	
Grades 1–2		0.88 (0.48–1.62)	0.678	0.86 (0.35–2.12)	0.741	0.76 (0.44–1.30)	0.309	0.67 (0.31–1.44)	0.304
Grades 3–4		0.60 (0.35–1.03)	0.063	0.82 (0.33–2.00)	0.662	0.56 (0.35–0.89)	0.014	0.66 (0.31–1.38)	0.267
*Thrombocytopenia*	437								
Grade 0		1.00		1.00		1.00		1.00	
Grades 1–2		0.56 (0.37–0.84)	0.005	0.49 (0.27–0.87)	0.016	0.58 (0.41–0.83)	0.003	0.50 (0.30–0.83)	0.008
Grades 3–4		0.59 (0.42–0.83)	0.003	0.63 (0.36–1.13)	0.119	0.59 (0.43–0.80)	0.001	0.58 (0.35–0.96)	0.035
*Histological response*^b^	351								
Poor		1.00		1.00		1.00		1.00	
Good		0.42 (0.28–0.62)	<0.001	0.42 (0.27–0.65)	<0.001	0.38 (0.27–0.54)	<0.001	0.36 (0.25–0.54)	<0.001
*Surgery type*^b^	506								
Amputation		1.00		1.00		1.00		1.00	
Limb salvage		0.80 (0.59–1.07)	0.130	0.81 (0.51–1.28)	0.367	0.84 (0.65–1.10)	0.214	0.84 (0.55–1.27)	0.407
*Timeliness of surgery*^b^	505								
On-time		1.00		1.00		1.00		1.00	
Early		1.67 (0.98–2.87)	0.060	0.91 (0.28–3.01)	0.878	1.61 (0.96–2.70)	0.070	0.77 (0.25–2.41)	0.654
Late		0.89 (0.67–1.18)	0.416	0.97 (0.67–1.41)	0.886	1.00 (0.78–1.29)	0.981	1.01 (0.73–1.40)	0.972

Models stratified by trial (except for year of randomisation in univariate models). Toxicity grade = maximum recorded over all cycles for each category. Location of tumour: proximal = proximal humerus/femur; distal = all other sites. Surgery type: amputation includes rotationplasty and disarticulation. Timeliness of surgery: on-time = between 3 d earlier and 10 d later than specified in protocol; early = more than 3 d earlier than specified; late = more than 10 d later than specified.

a Not included in multivariate model due to overlap with other variables.

b Timed from date of surgery (includes multivariate model).

## Appendix A Supplementary data

Supplementary data This document file contains supplementary tables.
