# Supplementary material for: Presence of chemotherapy-induced toxicity predicts improved survival in patients with localised extremity osteosarcoma treated with doxorubicin and cisplatin: A report from the European Osteosarcoma Intergroup
Source: Eur J Cancer. 2012 Mar;48(5):703–12. doi: 10.1016/j.ejca.2011.09.012 (PMC3657154; doi:10.1016/j.ejca.2011.09.012)
Supplement: Supplementary data — This document file contains supplementary tables. [file mmc1.doc]

**SUPPLEMENTARY DATA**

**Patient demographics and clinical characteristics, by trial**

|  | **Trial** | | | |
| --- | --- | --- | --- | --- |
|  | **BO02 (*n*=89)** | **BO03 (*n*=199)** | **BO06 (*n*=245)** | **Total (*n*=533)** |
| *Collaborative group* |  |  |  |  |
| MRC | 57 (64%) | 135 (68%) | 142 (58%) | 334 (63%) |
| EORTC | 32 (36%) | 64 (32%) | 103 (42%) | 199 (37%) |
|  |  |  |  |  |
| *Geographical location* |  |  |  |  |
| UK/Ireland | 57 (64%) | 133 (67%) | 113 (46%) | 303 (57%) |
| Mainland Europe | 30 (34%) | 35 (18%) | 71 (29%) | 136 (26%) |
| Others^a^ | 2 (2%) | 31 (16%) | 61 (25%) | 94(18%) |
|  |  |  |  |  |
| *Age at randomisation* |  |  |  |  |
| Median (years) | 16 (13–19) | 16 (13–20) | 15 (11–18) | 15 (12–19) |
| Min–Max | 3–40 | 4–36 | 3–37 | 3–40 |
| 0–10 years | 6 (7%) | 32 (16%) | 55 (22%) | 93 (17%) |
| 11–15 years | 32 (36%) | 59 (30%) | 90 (37%) | 181 (34%) |
| 16–20 years | 36 (41%) | 72 (36%) | 64 (26%) | 172 (32%) |
| 21–25 years | 11 (12%) | 25 (13%) | 16 (7%) | 52 (10%) |
| ≥26 years | 4 (5%) | 11 (6%) | 20 (8%) | 35 (7%) |
| Missing | 0 (n/a) | 0 (n/a) | 0 (n/a) | 0 (n/a) |
|  |  |  |  |  |
| *Sex* |  |  |  |  |
| Male | 48 (54%) | 131 (66%) | 144 (60%) | 323 (61%) |
| Female | 41 (46%) | 68 (34%) | 98 (41%) | 207 (39%) |
| Missing | 0 (n/a) | 0 (n/a) | 3 (n/a) | 3 (n/a) |
|  |  |  |  |  |
| *Site of tumour* |  |  |  |  |
| Femur | 51 (57%) | 113 (57%) | 143 (59%) | 307 (58%) |
| Tibia | 26 (29%) | 50 (25%) | 60 (25%) | 136 (26%) |
| Fibula | 6 (7%) | 9 (5%) | 13 (5%) | 28 (5%) |
| Humerus | 6 (7%) | 24 (12%) | 22 (9%) | 52 (10%) |
| Radius | 0 (0%) | 3 (2%) | 3 (1%) | 6 (1%) |
| Missing | 0 (n/a) | 0 (n/a) | 4 (n/a) | 4 (n/a) |
|  |  |  |  |  |
| *Location of tumour* |  |  |  |  |
| Proximal^b^ | 11 (12%) | 27 (14%) | 31 (13%) | 69 (13%) |
| Distal^c^ | 78 (88%) | 171 (86%) | 209 (87%) | 458 (87%) |
| Missing | 0 (n/a) | 1 (n/a) | 5 (n/a) | 6 (n/a) |
|  |  |  |  |  |
| *Classification of sarcoma* |  |  |  |  |
| Common-type | 73 (83%) | 132 (66%) | 126 (59%) | 331 (66%) |
| Chondroblastic | 4 (5%) | 26 (13%) | 25 (12%) | 55 (11%) |
| Fibroblastic | 6 (7%) | 23 (12%) | 9 (4%) | 38 (8%) |
| Osteoclast rich | 0 (0%) | 4 (2%) | 2 (1%) | 6 (1%) |
| Anaplastic | 3 (3%) | 4 (2%) | 9 (4%) | 16 (3%) |
| Small cell | 1 (1%) | 2 (1%) | 3 (1%) | 6 (1%) |
| Telangiectatic | 0 (0%) | 4 (2%) | 22 (10%) | 26 (5%) |
| Other | 1 (1%) | 4 (2%) | 17 (8%) | 22 (4%) |
| Missing | 1 (n/a) | 0 (n/a) | 32 (n/a) | 33 (n/a) |

Data are number (%) or median (IQR).

^a^ Other geographical location = South America, South Africa, Saudi Arabia, Canada, New Zealand.

^b^ Proximal = Proximal humerus/femur.

^c^ Distal = All other sites.

**Treatment details, response and toxicity by trial**

|  | **Trial** | | | |
| --- | --- | --- | --- | --- |
|  | **BO02 (*n*=89)** | **BO03 (*n*=199)** | **BO06 (*n*=245)** | **Total (*n*=533)** |
| *Reason off-study* |  |  |  |  |
| Treatment completed | 67 (75%) | 167 (84%) | 189 (78%) | 423 (80%) |
| Disease progression | 5 (6%) | 13 (7%) | 11 (5%) | 29 (6%) |
| Excessive toxicity | 12 (14%) | 11 (6%) | 12 (5%) | 35 (7%) |
| Treatment refusal | 1 (1%) | 3 (2%) | 12 (5%) | 16 (3%) |
| Other | 4 (5%) | 4 (2%) | 17 (7%) | 25 (5%) |
| Missing | 0 (n/a) | 1 (n/a) | 4 (n/a) | 5 (n/a) |
|  |  |  |  |  |
| *Type of surgery* |  |  |  |  |
| Amputation | 39 (44%) | 51 (26%) | 57 (26%) | 147 (29%) |
| Limb salvage | 50 (56%) | 145 (74%) | 164 (74%) | 359 (71%) |
| Missing | 0 (n/a) | 1 (n/a) | 24 (n/a) | 25 (n/a) |
|  |  |  |  |  |
| *Histological response* |  |  |  |  |
| Poor | 7 (41%) | 96 (69%) | 125 (64%) | 228 (65%) |
| Good | 10 (59%) | 43 (31%) | 70 (36%) | 123 (35%) |
| Missing | 72 (n/a) | 60 (n/a) | 50 (n/a) | 182 (n/a) |
|  |  |  |  |  |
| *Max oral mucositis grade* |  |  |  |  |
| Grade 0 | 39 (48%) | 78 (39%) | 63 (27%) | 180 (35%) |
| Grade 1–2 | 32 (39%) | 81 (41%) | 110 (46%) | 223 (43%) |
| Grade 3–4 | 11 (13%) | 40 (20%) | 64 (27%) | 115 (22%) |
| Missing | 7 (n/a) | 0 (n/a) | 8 (n/a) | 15 (n/a) |
|  |  |  |  |  |
| *Max nausea/vomiting grade* |  |  |  |  |
| Grade 0 | 1 (1%) | 5 (3%) | 9 (4%) | 15 (3%) |
| Grade 1–2 | 21 (25%) | 46 (23%) | 115 (49%) | 182 (35%) |
| Grade 3–4 | 61 (74%) | 148 (74%) | 113 (48%) | 322 (62%) |
| Missing | 6 (n/a) | 0 (n/a) | 8 (n/a) | 14 (n/a) |
|  |  |  |  |  |
| *Max cardiac grade^a^* |  |  |  |  |
| Grade 0 | 72 (88%) | 188 (95%) | 204 (86%) | 464 (90%) |
| Grade 1–2 | 10 (12%) | 11 (6%) | 27 (11%) | 48 (9%) |
| Grade 3–4 | 0 (0%) | 0 (0%) | 6 (3%) | 6 (1%) |
| Missing | 7 (n/a) | 0 (n/a) | 8 (n/a) | 15 (n/a) |
|  |  |  |  |  |
| *Max infection grade* |  |  |  |  |
| Grade 0 | 47 (58%) | 79 (40%) | 70 (30%) | 196 (38%) |
| Grade 1–2 | 21 (26%) | 78 (39%) | 102 (43%) | 201 (39%) |
| Grade 3–4 | 13 (16%) | 42 (21%) | 65 (27%) | 120 (23%) |
| Missing | 8 (n/a) | 0 (n/a) | 8 (n/a) | 16 (n/a) |
|  |  |  |  |  |
| *Max neurological grade* |  |  |  |  |
| Grade 0 | 69 (85%) | 176 (88%) | 213 (90%) | 458 (89%) |
| Grade 1–2 | 12 (15%) | 22 (11%) | 24 (10%) | 58 (11%) |
| Grade 3–4 | 0 (0%) | 1 (1%) | 0 (0%) | 1 (0%) |
| Missing | 8 (n/a) | 0 (n/a) | 8 (n/a) | 16 (n/a) |
|  |  |  |  |  |
| *Max leucopenia grade* |  |  |  |  |
| Grade 0 | n/a | 17 (9%) | 11 (5%) | 28 (6%) |
| Grade 1–2 | n/a | 32 (16%) | 34 (14%) | 66 (15%) |
| Grade 3–4 | n/a | 150 (75%) | 193 (81%) | 343 (79%) |
| Missing | 89 (n/a) | 0 (n/a) | 7 (n/a) | 96 (n/a) |
|  |  |  |  |  |
| *Max thrombocytopenia grade* |  |  |  |  |
| Grade 0 | n/a | 50 (25%) | 45 (19%) | 95 (22%) |
| Grade 1–2 | n/a | 58 (29%) | 56 (24%) | 114 (26%) |
| Grade 3–4 | n/a | 91 (46%) | 137 (58%) | 228 (52%) |
| Missing | 89 (n/a) | 0 (n/a) | 7 (n/a) | 96 (n/a) |

Data are number (%).

^a^As recorded during chemotherapy only.

**Univariate and multivariate models for odds of a good histological response**

|  | Univariate models | | | Multivariate model (n=325) | |
| --- | --- | --- | --- | --- | --- |
|  | *n* | Odds ratio  (95% CI) | *p* value | Odds ratio  (95% CI) | *p* value |
| *Collaborative group* | 351 |  |  |  |  |
| MRC |  | 1.00 |  | 1.00 |  |
| EORTC |  | 0.92 (0.57–1.48) | 0.738 | 0.50 (0.13–1.88) | 0.304 |
| *Geographical Location* | 351 |  |  |  |  |
| UK/Ireland |  | 1.00 |  | 1.00 |  |
| Mainland Europe |  | 0.91 (0.52–1.59) | 0.734 | 2.00 (0.42–9.47) | 0.384 |
| Other countries |  | 1.60 (0.88–2.92) | 0.126 | 2.94 (0.93–9.30) | 0.067 |
| *Age group* | 351 |  |  |  |  |
| ≤10 years |  | 1.00 |  | 1.00 |  |
| 11–15 years |  | 0.78 (0.43–1.43) | 0.424 | 0.66 (0.33–1.32) | 0.244 |
| 16–20 years |  | 0.65 (0.34–1.22) | 0.178 | 0.57 (0.27–1.20) | 0.140 |
| 21–25 years |  | 1.07 (0.44–2.59) | 0.878 | 0.99 (0.37–2.65) | 0.987 |
| ≥26 years |  | 0.25 (0.07–0.91) | 0.036 | 0.18 (0.04–0.72) | 0.015 |
| *Gender* | 351 |  |  |  |  |
| Male |  | 1.00 |  | 1.00 |  |
| Female |  | 0.92 (0.58–1.45) | 0.710 | 0.94 (0.54–1.61) | 0.808 |
| *Site of tumour^a^* | 351 |  |  |  |  |
| Femur |  | 1.00 |  |  |  |
| Tibia |  | 1.53 (0.92–2.55) | 0.104 |  |  |
| Fibula |  | 1.09 (0.41–2.89) | 0.860 |  |  |
| Humerus |  | 0.87 (0.40–1.88) | 0.724 |  |  |
| Other |  | 0.54 (0.06–4.91) | 0.582 |  |  |
| *Location of tumour* | 351 |  |  |  |  |
| Proximal femur/humerus |  | 1.00 |  | 1.00 |  |
| Other site |  | 1.52 (0.77–3.00) | 0.233 | 1.44 (0.67–3.07) | 0.351 |
| *Type of osteosarcoma* | 345 |  |  |  |  |
| Common-type |  | 1.00 |  | 1.00 |  |
| Chondroblastic |  | 0.31 (0.12–0.77) | 0.012 | 0.28 (0.10–0.77) | 0.014 |
| Fibroblastic |  | 1.50 (0.65–3.47) | 0.341 | 1.87 (0.71–4.96) | 0.206 |
| Anaplastic |  | 0.99 (0.28–3.51) | 0.993 | 1.47 (0.37–5.80) | 0.580 |
| Telangiectatic |  | 1.82 (0.73–4.52) | 0.200 | 1.84 (0.69–4.95) | 0.224 |
| Other |  | 0.37 (0.14–1.03) | 0.058 | 0.49 (0.17–1.45) | 0.199 |
| *Oral mucositis* | 349 |  |  |  |  |
| Grade 0 |  | 1.00 |  | 1.00 |  |
| Grade 1–2 |  | 1.32 (0.82–2.11) | 0.257 | 1.48 (0.85–2.57) | 0.168 |
| Grade 3–4 |  | 1.18 (0.57–2.46) | 0.655 | 1.09 (0.43–2.74) | 0.858 |
| *Nausea/vomiting* | 351 |  |  |  |  |
| Grade 0 |  | 1.00 |  | 1.00 |  |
| Grade 1–2 |  | 1.49 (0.55–4.04) | 0.429 | 1.80 (0.57–5.71) | 0.318 |
| Grade 3–4 |  | 1.56 (0.58–4.21) | 0.376 | 1.65 (0.52–5.22) | 0.395 |
| *Infection* | 349 |  |  |  |  |
| Grade 0 |  | 1.00 |  | 1.00 |  |
| Grade 1–2 |  | 0.90 (0.55–1.47) | 0.676 | 1.07 (0.60–1.92) | 0.824 |
| Grade 3–4 |  | 1.15 (0.58–2.29) | 0.690 | 1.09 (0.44–2.67) | 0.857 |
| *Cardiac* | 349 |  |  |  |  |
| Grade 0 |  | 1.00 |  | 1.00 |  |
| Grade 1–2 |  | 1.46 (0.51–4.23) | 0.481 | 1.33 (0.37–4.75) | 0.663 |
| Grade 3–4 |  | n/a | n/a | n/a | n/a |
| *Neurological* | 350 |  |  |  |  |
| Grade 0 |  | 1.00 |  | 1.00 |  |
| Grade 1–2 |  | 0.78 (0.26–2.36) | 0.664 | 0.79 (0.20–3.10) | 0.738 |
| Grade 3–4 |  | n/a | n/a | n/a | n/a |
| *Leucopenia* | 334 |  |  |  |  |
| Grade 0 |  | 1.00 |  | 1.00 |  |
| Grade 1–2 |  | 1.58 (0.64–3.88) | 0.319 | 1.35 (0.50–3.66) | 0.553 |
| Grade 3–4 |  | 1.45 (0.61–3.41) | 0.401 | 1.28 (0.45–3.61) | 0.640 |
| *Thrombocytopenia* | 334 |  |  |  |  |
| Grade 0 |  | 1.00 |  | 1.00 |  |
| Grade 1–2 |  | 0.91 (0.52–1.57) | 0.726 | 0.81 (0.41–1.60) | 0.549 |
| Grade 3–4 |  | 1.00 (0.57–1.75) | 0.991 | 1.00 (0.46–2.16) | 0.996 |
| *Type of surgery^b^* | 351 |  |  |  |  |
| Amputation |  | 1.00 |  | 1.00 |  |
| Limb salvage |  | 1.16 (0.69–1.94) | 0.578 | 1.20 (0.61–2.38) | 0.600 |
| *Timeliness of surgery^b^* | 340 |  |  |  |  |
| On-time |  | 1.00 |  | 1.00 |  |
| Early |  | n/a | n/a | n/a | n/a |
| Late |  | 1.23 (0.78–1.94) | 0.375 | 1.14 (0.67–1.94) | 0.622 |

Models stratified by trial. Toxicity grade = maximum recorded over pre-operative cycles. Location of tumour: proximal = proximal humerus/femur; distal = all other sites**.** Surgery type: amputation includes rotationplasty and disarticulation. Timeliness of surgery: on-time = between 3 days earlier and 10 days later than specified in protocol; early = more than 3 days earlier than specified; late = more than 10 days later than specified.

^a^ Not included in multivariate model due to overlap with other variables.

^b^ Timed from date of surgery (includes multivariate model).
